# Differences in Cerebral Tissue Oxygenation in Preterm Neonates Receiving Adult or Cord Blood Red Blood Cell Transfusions

**DOI:** 10.1001/jamanetworkopen.2023.41643

**Published:** 2023-11-07

**Authors:** Claudio Pellegrino, Patrizia Papacci, Flavia Beccia, Francesca Serrao, Giulia Vanina Cantone, Giorgio Cannetti, Carmen Giannantonio, Giovanni Vento, Luciana Teofili

**Affiliations:** 1 Department of Image, Radiation Therapy, Oncology and Hematology Diagnosis, Fondazione Policlinico Universitario A. Gemelli IRCCS, Hospitalization and Health Care, Roma, Italy; 2 Department of Radiological and Hematological Sciences, Università Cattolica del Sacro Cuore, Rome, Italy; 3 Department of Woman and Child Health and Public Health, Fondazione Policlinico Universitario A. Gemelli IRCCS, Hospitalization and Health Care, Rome, Italy; 4 Department of Life Science and Public Health, Università Cattolica del Sacro Cuore, Rome, Italy

## Abstract

**Importance:**

Repeated transfusions in preterm neonates with anemia of prematurity replace fetal hemoglobin (HbF) with adult Hb (HbA), which has a low oxygen affinity. The reduction of HbF is associated with a higher incidence of retinopathy of prematurity (ROP).

**Objective:**

To assess whether HbF and HbA are differently associated with cerebral tissue oxygenation in preterm neonates.

**Design, Setting, and Participants:**

This cohort study was a single-center, pilot study on cerebral oxygenation kinetics in preterm neonates with a gestational age between 24.0 weeks and 27.9 weeks who were admitted to the neonatal intensive care unit of Policlinico Universitario A. Gemelli IRCCS from December 27, 2021, to May 15, 2023. This study was ancillary to the ongoing, double-blind, multicenter Umbilical or Adult Donor Red Blood Cells in Extremely Low Gestational Age Neonates and Retinopathy of Prematurity (BORN) randomized clinical trial. The BORN trial outcome was ROP severity in neonates randomized to receive standard packed red blood cell (PRBC) transfusions obtained from RBCs of adult donors (A-RBCs) or from cord blood (CB-RBCs). According to standard procedures at the institute’s neonatal intensive care unit, patients concurrently received continuous cerebral near-infrared spectroscopy (NIRS) monitoring. This cohort study was not prespecified in the trial protocol.

**Exposure:**

Transfusion with A-RBCs or CB-RBCs.

**Main Outcomes and Measures:**

The main outcome was the kinetics of cerebral regional oxygen saturation (crSO_2_) and cerebral fraction of tissue oxygen extraction (cFTOE) associated with A-RBC or CB-RBC transfusions. Cerebral NIRS monitoring was performed by neonatologists and nurses, who were blinded to the PRBC type. The NIRS monitoring was conducted starting with the blood product order, during transfusion, and for the subsequent 24 hours after transfusion completion. The mean treatment effects of A-RBCs or CB-RBCs were quantified using a linear mixed model for repeated measures.

**Results:**

Of 23 randomized neonates, 17 (11 male [64.7%]; median gestational age at birth, 25.6 weeks [IQR, 25.3-26.1 weeks]) with a median birth weight of 840 g (IQR, 580-900 g) were included in the study; NIRS was evaluated for 42 transfusion episodes, of which 22 were A-RBCs and 20 were CB-RBCs. Globally considering all posttransfusion time points, the overall crSO_2_ covariate-adjusted mean after CB-RBC transfusions was 5.27% lower (95% CI, 1.20%-9.34%; *P* = .01) than that after A-RBC transfusions, while the cFTOE after CB-RBC transfusions was 6.18% higher (95% CI, 1.66%-10.69%; *P* = .009) than that after A-RBCs.

**Conclusions and Relevance:**

The findings of this cohort study suggest that A-RBC transfusions may be associated with more oxygen delivery to cerebral tissues of preterm neonates than transfusions from CB-RBCs. This finding may explain the previously observed association between low HbF and high ROP risk. It also suggests that use of CB to meet the RBC transfusion needs of neonates with a gestational age of less than 28 weeks may protect cerebral tissues from overexposure to oxygen.

## Key Points

**Question:**

Are fetal and adult hemoglobin differently associated with cerebral tissue oxygenation kinetics of preterm neonates with anemia of prematurity?

**Findings:**

In this cohort study of 23 randomized neonates, cerebral near-infrared spectroscopy was monitored before and after 42 packed red blood cell (PRBC) transfusions in 17 preterm neonates, of which 22 units were standard PRBCs from adult donors and 20 were PRBCs obtained from allogeneic cord blood. A higher regional oxygen saturation and a lower fraction of oxygen extraction were recorded after adult transfusions compared with cord blood transfusions, suggesting that adult hemoglobin was associated with an overexposure to oxygen in cerebral tissue.

**Meaning:**

The findings suggest that cord blood transfusions in neonates with anemia of prematurity may help prevent inappropriate oxygen exposure of cerebral tissue.

## Teaser

This cohort study assessed whether adult or allogeneic cord blood packed red blood cell (RBC) transfusions were associated with different cerebral tissue oxygenation kinetics of 17 preterm neonates.

## Introduction

Near-infrared spectroscopy (NIRS) offers a noninvasive, real-time monitoring method of tissue oxygenation, shedding light on the delicate balance between oxygen delivery and consumption.^1^ The NIRS software calculates regional oxygen saturation (rSO_2_) relative concentrations of oxygenated hemoglobin (Hb) and deoxygenated Hb at a tissue depth of 1 cm to 2 cm. Combining values of rSO_2_ and arterial oxygen saturation, the regional fraction of tissue oxygen extraction (FTOE) can be computed, which is a measure of the amount of oxygen extracted by the tissue and an estimate of the balance between local oxygen delivery and consumption.^2^

NIRS monitoring has been useful among preterm neonates with a variety of clinical conditions and has the potential to guide clinical interventions, including packed red blood cell (PRBC) transfusion.^3^ Liem et al^4^ reported an improvement of cerebral oxygenation after PRBC transfusions in a cohort of preterm newborns undergoing NIRS monitoring. The increase in oxygenated Hb concentration was larger than that in deoxygenated Hb, reflecting a parallel increase in arterial oxygen content and a reduction in the concentration of fetal Hb (HbF), which was associated with a decrease of Hb oxygen affinity. Subsequent studies supported an increase in cerebral rSO_2_ (crSO_2_) and a decrease in cerebral FTOE (cFTOE) in the 24 hours after transfusion, with greater changes in neonates with lower baseline Hb and crSO_2_ levels.^5,6,7^

The progressive reduction of HbF in preterm neonates with anemia of prematurity has been associated with retinopathy of prematurity (ROP) and other unfavorable outcomes.^8,9^ To raise the Hb concentration without depleting the physiological HbF reservoir, we explored a transfusion strategy based on erythrocyte concentrates obtained from allogeneic cord blood RBCs (CB-RBCs).^10^ How this approach may be associated with cerebral oxygenation is presently unknown. In this study, we investigated cerebral oxygenation kinetics after transfusion of CB-RBC concentrates or standard PRBC units obtained from adult donors.

## Methods

### Study Design and Population

This single-center, prospective, pilot cohort study evaluated cerebral oxygenation kinetics in preterm neonates receiving standard PRBC units—adult RBCs (A-RBCs) or CB-RBCs. The present study was an ancillary, nonprespecified study of the Umbilical or Adult Donor Red Blood Cells in Extremely Low Gestational Age Neonates and Retinopathy of Prematurity (BORN) trial^11^; the study population included preterm neonates enrolled at the neonatal intensive care unit of the Fondazione Policlinico Universitario A. Gemelli IRCCS, Hospitalization and Health Care (IRCCS) from December 27, 2021, to May 15, 2023. The present study followed the Strengthening the Reporting of Observational Studies in Epidemiology (STROBE) reporting guideline for cohort studies. The BORN trial is an ongoing, double-blind, multicenter randomized clinical trial that was designed to investigate whether transfusion of preterm neonates with CB-RBCs instead of A-RBCs reduces the severity of ROP.^11^ The BORN trial was approved by the ethics committee of the Fondazione Policlinico Universitario A. Gemelli IRCCS. Consent for this study was waived because it included secondary data gathered in the BORN trial. Inclusion criteria comprised gestational age between 24.0 weeks and 27.9 weeks and signed, written informed consent of parents. Exclusion criteria included any of the following: maternal-fetal immunization, hydrops fetalis, major congenital malformations associated or not with genetic syndromes, previous transfusions, hemorrhage at birth, congenital infections, and the health care team deeming it inappropriate to approach the neonate’s family for informed consent. Patients received A-RBC or CB-RBC transfusions according to the BORN trial group assignment and NIRS monitoring according to standard care in the neonatal intensive care unit. The main outcome was cerebral tissue oxygenation in neonates receiving A-RBC or CB-RBC transfusions.

### Blood Products and Transfusion Procedures

According to the BORN trial design, patients were randomized 1:1 to receive standard A-RBCs (group A, comparator) or CB-RBCs (group B, intervention). Units of RBCs were ABO-RhD blood group matched; if the ABO-RhD blood group–matched CB-RBC unit was unavailable, patients in group B received an A-RBC unit. CB-RBCs were obtained from CB solidaristic donations at the public CB bank of the Fondazione Policlinico Universitario A. Gemelli IRCCS. The units not suitable for transplant for a low, total nucleated cell content were used. Cord blood units were processed as previously described.^11^ Units were γ irradiated, and neonates received a PRBC dose of 20 mL/kg by continuous infusion during a 4-hour period (BD Alaris pump infusion sets). The transfusion threshold was individually defined depending on the venous hematocrit (Hct) level, age, and presence of symptoms that suggested inadequate tissue oxygenation.^12^ The peripheral arterial oxygen saturation (SpO_2_) target was set according to postmenstrual age and was never superior to 95%.

### Cerebral Oxygenation Monitoring and NIRS Data Alignment

**Figure 1.**
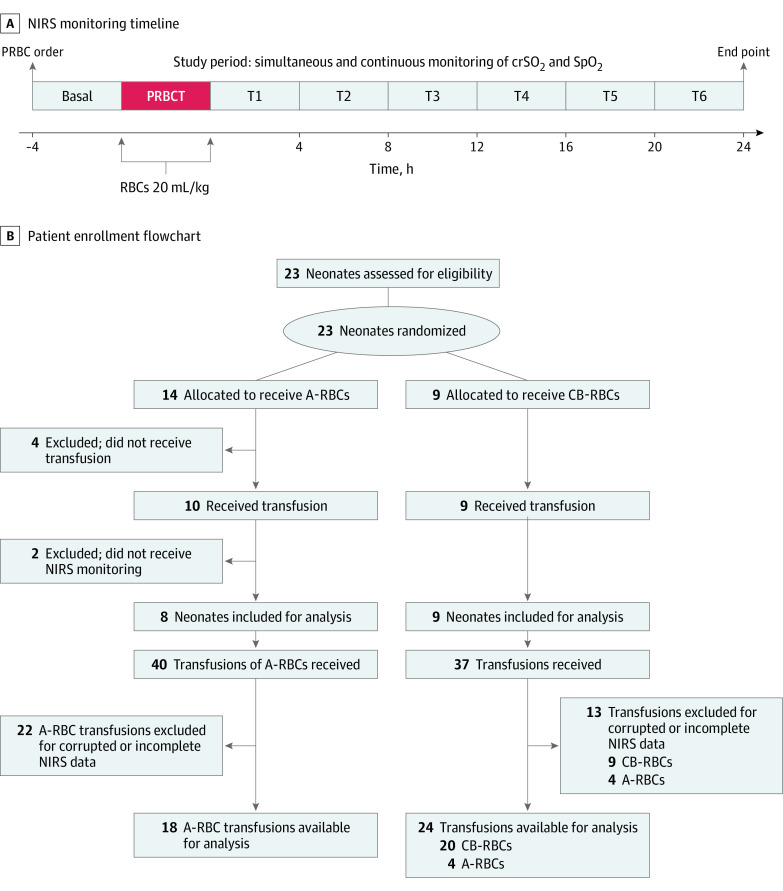
Timeline of Near-Infrared Spectroscopy (NIRS) Monitoring and Flowchart of Study Population Selection A-RBCs indicates adult red blood cells; CB, cord blood; crSO_2_, cerebral regional oxygen saturation; PRBC, packed RBC; PRBCT, PRBC transfusion; SpO_2_, peripheral arterial oxygen saturation; and T, time point.

Cerebral NIRS monitoring was performed by neonatologists (P.P., F.S., G.V.C., C.G.) and nurses, who were blinded to the PRBC typeThe crSO_2_ was measured through a 2-wavelength NIRS monitor (Covidien INVOS 5100C; Medtronic). The start and end times of the transfusion and periods of care (eg, nurturing), neonate handling, and other clinically relevant records were electronically annotated in real time (Digistat; Ascom). The NIRS recording started once the transfusion was ordered and continued during PRBC administration and for the 24 hours after its completion (Figure 1A). Oxygenation data were downloaded in real time from the NIRS monitor to a laptop computer at a sampling rate of 0.25 Hz and aligned along the time axis using the INVOS Monitoring System Analytics Tool, version 1.2.1 (Medtronic). The SpO_2_ was recorded concurrently through a pulse oximeter (Nellcor; Medtronic) and exported (Nellcor Analytics Tool; Medtronic) for the calculation of cFTOE using the following formula: (SpO_2_ − crSO_2_)/SpO_2_. Records pertaining to NIRS were manually aligned to the start and to the end times of the transfusions, removing corrupted or artifactual data.^13,14^ Interpolation strategies were not applied to avoid introduction of errors.^15^ Hourly means and 95% CIs were obtained and examined at the time points indicated in Figure 1A.

### Data Collection

Patients’ data were gathered through the REDCap database hosted at the Fondazione Policlinico Universitario A. Gemelli IRCCS and retrieved on May 31, 2023, for the prespecified interim analysis of the BORN trial.^11^ Data at birth included demographics, gestational age, weight, maternal obstetrical history, hematologic parameters (Hb, Hct, and HbF values, expressed as the percentage of total Hb), Apgar score at 1 minute and 5 minutes, and the Clinical Risk Index for Babies II (CRIB II) score (scores range from 0 to 27, with lower scores indicating a better prognosis).^16^ The following variables potentially affecting cerebral hemodynamic and oxygenation kinetics at transfusion were also retrieved: postmenstrual age; body weight; fraction of inspired oxygen; type of ventilatory support; lactate; Hb, Hct, and HbF values; presence of hemodynamically significant patent ductus arteriosus; presence of intraventricular hemorrhage^17^; and resistive indexes of the anterior cerebral artery as a marker of cerebral blood flow and cerebrovascular reactivity.^18^

### Statistical Analysis

In descriptive analyses, continuous variables were expressed as median and IQR and categorical variables as absolute and relative frequencies. To compare continuous variables, we used the Mann-Whitney test or the Kruskal-Wallis test as appropriate; for categorical variables, we used the Fisher exact test or the χ^2^ test as appropriate. Potential correlation of pretransfusion variables and basal crSO_2_ or cFTOE was assessed by the Pearson product-moment correlation analysis. The mean treatment effects (95% CI) of A-RBC and CB-RBC transfusions were quantified using the linear mixed model for repeated measures (MMRM), considering crSO_2_ or cFTOE as dependent variables. Treatment effects were calculated at 4-hour intervals up to 24 hours after transfusion. The models were adjusted for baseline values of the measured outcome and for postmenstrual age at transfusion. The same covariates and outcomes were adopted in a restricted analysis including only first transfusion episodes. The models considered the possible association of multiple transfusions with different types of PRBC units. Sensitivity analyses were performed using the unadjusted models and models restricted to multiple transfusions. Selection of covariance structure for the model was based on the minimum Akaike information criteria. Normal distribution of model residuals was confirmed with a histogram. Differences with 2-sided *P* ≤ .05 were considered significant. Statistical analyses were performed using SPSS Statistics, version 29 (IBM Corp) (eAppendix in Supplement 1) and Stata, version 17 (StataCorp LLC).

## Results

### Patient Population and Characteristics at Transfusion

A flowchart of the 23 patients potentially eligible, the 6 excluded, and the 17 analyzed is provided in Figure 1B. Table 1 summarizes baseline parameters at birth of the neonates’ cohort included in the final analysis. Of 17 neonates, 6 (35.3%) were female, 11 (64.7%) were male, and 3 were twins (17.6%), comprising 1 couple and 1 single neonate whose twin was excluded due to death shortly after birth. Median gestational age at birth was 25.6 weeks (IQR, 25.3-26.1 weeks), and median birth weight was 840 g (IQR, 580-900 g). The median value of Hb was 14.6 g/dL (IQR 10.6-18.6 g/dL) (to convert from grams per deciliter to grams per liter, multiply by 10), of central Hct was 43.0% (IQR, 38.4%-48.3%), and of HbF was 95.6% (IQR, 92.3%-96.2%). The basal CRIB II score was 11 (IQR, 8-14) with an anticipated overall mortality rate of 17.9% (IQR, 5.3%-45.6%). Nine patients were randomized to CB-RBC group B and 8 patients to A-RBC group A. All patients in group A (comparator group) received only A-RBCs. Conversely, in group B (intervention group), 2 patients received mixed transfusion support due to unavailability of matched CB-RBC units (3 A-RBC and 5 CB-RBC units in 1 patient and 1 A-RBC and 3 CB-RBC units in another patient).

**Table 1.** Demographics and Clinical and Laboratory Characteristics of the Study Population

Characteristic	Patients (N = 17)^a^
Sex	
Female	6 (35.3)
Male	11 (64.7)
Twin	3 (17.6)
Basal obstetric pathology	
Placental abruption	4 (23.5)
Placenta previa	2 (11.8)
Eclampsia	2 (11.8)
Chorioamnionitis	17 (100)
PROM	8 (47.1)
IUGR	3 (17.6)
Antenatal steroids	7 (41.2)
Gestational age at birth, wk	25.6 (25.3-26.1)
Birth weight, g	840 (580-900)
Apgar score^b^	
1 min	6 (3-9)
5 min	8 (7-9)
Basal CRIB II score^c^	8 (4-14)
Anticipated mortality	17.9 (5.3-45.6)
Hemoglobin, g/dL	14.6 (10.6-18.6)
Central hematocrit, %	43.0 (38.4-48.3)
Peripheral hematocrit, %	46.0 (33.0-52.0)
Fetal hemoglobin, %	95.6 (92.3-96.2)

Abbreviations: CRIB II, Clinical Risk Index for Babies II; IUGR, intrauterine growth restriction; PROM, premature rupture of membranes.

SI conversion factor: To convert hemoglobin from grams per deciliter to grams per liter, multiply by 10.

^a^ Categorical variables (sex, twin, basal obstetric pathology, and antenatal steroids) are reported as No. (%). Continuous variables (gestational age at birth, birth weight, Apgar score, basal CRIB II score, and hematologic parameters at birth) are reported as median (IQR).

^b^ Scores range from 0 to 10, with higher scores indicating a healthier assessment 1 minute and 5 minutes after birth.

^c^ Scores range from 0 to 27, with lower scores indicating a better prognosis.

The 17 neonates included in the analysis received 42 total transfusions, of which 22 were A-RBCs and 20 were CB-RBCs. For each patient, the type of blood product and the progressive number of the transfusion event are detailed in eTable 1 in Supplement 1. Table 2 shows recipients’ characteristics at the specific transfusion events according to the blood product received. The volume of the infused product was comparable, and increases in Hct level were similar with both A-RBC and CB-RBC transfusions. Median postmenstrual age at transfusion was 27.4 weeks (IQR, 26.3-28.1 weeks) in the A-RBC group and 29.0 weeks (IQR, 26.6-31.1 weeks) in the CB-RBC group (*P* = .06); subsequent analysis was adjusted for this potential confounder. There were no differences between A-RBCs and CB-RBCs in pretransfusion levels of Hb, Hct, and HbF; lactate; oxygen supplementation; and type of ventilatory support (Table 2). Concomitant hemodynamically significant patent ductus arteriosus and intraventricular hemorrhage were recorded in association with A-RBC transfusions (hemodynamically significant patent ductus arteriosus, 54.6% and intraventricular hemorrhage, 54.6%) and with CB-RBC transfusions (hemodynamically significant patent ductus arteriosus, 35.0% and intraventricular hemorrhage, 45.0%). Finally, median resistive indexes of the anterior cerebral artery before A-RBC and CB-RBC transfusions were comparable (Table 2).

**Table 2.** Clinical and Laboratory Parameters in Neonates at the Time of Each Transfusion Event^a^

Parameter	A-RBC (n = 22)	CB-RBC (n = 20)	*P* value^b^
Postmenstrual age, wk	27.4 (26.3-28.1)	29.0 (26.6-31.1)	.06
Weight, g	855 (720-965)	980 (655-1250)	.09
Pretransfusion testing			
Hemoglobin, g/dL	10.0 (9.1-10.5)	10.4 (10.2-10.5)	.26
Hematocrit, %	30.0 (28.0-32.0)	30.8 (28.0-32.0)	.81
Fetal hemoglobin, %	50.9 (22.3-93.5)	91.8 (52.6-93.4)	.19
Lactate, mg/dL	13.06 (8.11-20.90)	12.16 (6.49-21.89)	.67
Ventilatory support			
None	0	2 (10.0)	.20
NIV or CPAP	3 (13.6)	5 (25.0)	
HFOV	6 (27.3)	6 (30.0)	
SIMV	13 (59.1)	7 (35.0)	
FiO_2_, %	0.4 (0.3-0.5)	0.3 (0.2-0.4)	.12
hs-PDA	12 (54.6)	7 (35.0)	.20
ACA-RI	0.8 (0.7-1.0)	0.8 (0.7-0.8)	.12
IVH grade at transfusion			
0	10 (45.4)	11 (55.0)	.25
I	3 (13.6)	1 (5.0)	
II	1 (4.6)	4 (20.0)	
III	8 (36.4)	4 (20.0)	
Previous RBC transfusions			
0	9 (40.9)	8 (40.0)	.51
1	3 (13.6)	3 (15.0)	
2	5 (22.7)	2 (10.0)	
≥3	5 (22.7)	7 (35.0)	
Basal crSO_2_, %	62.8 (50.5-67.8)	68.2 (62.6-76.2)	.05
Basal cFTOE, %	32.2 (26.2-44.0)	25.2 (18.2-34.8)	.07

Abbreviations: A-RBC, adult red blood cell; ACA-RI, resistive index of the anterior cerebral artery; CB-RBC, cord blood RBC; cFTOE, cerebral fraction of tissue oxygen extraction; CPAP, continuous positive airway pressure; crSO_2_, cerebral regional oxygen saturation; FiO_2_, fraction of inspired oxygen; HFOV, high-frequency oscillation ventilation; hs-PDA, hemodynamically significant patent ductus arteriosus; IVH, intraventricular hemorrhage; NIV, noninvasive ventilation; SIMV, synchronized intermittent mandatory ventilation.

SI conversion factors: To convert hemoglobin from grams per deciliter to grams per liter, multiply by 10; to convert lactate to mmol/L, multiply by 0.111.

^a^ Continuous variables (postmenstrual age, weight, pretransfusion testing, FiO_2_, ACA-RI, basal crSO_2_, and basal cFTOE) are reported as median (IQR). Categorical variables (ventilatory support, hs-PDA, IVH grade at transfusion, and previous RBC transfusions) are reported as No. (%).

^b^ Comparison of A-RBC and CB-RBC transfusions.

### Association of A-RBC and CB-RBC Transfusions With crSO_2_

**Figure 2.**
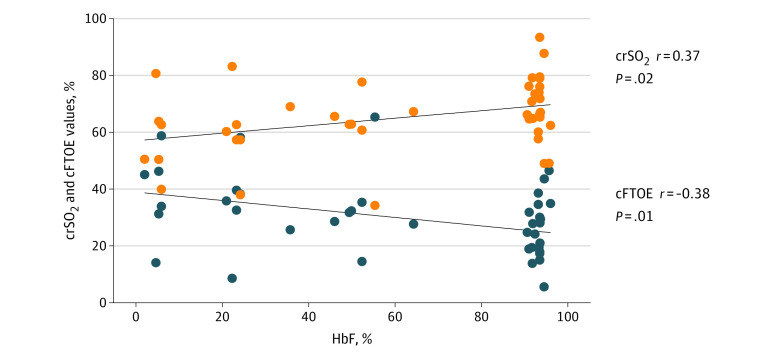
Correlation Between Pretransfusion Fetal Hemoglobin (HbF) and Baseline Cerebral Regional Oxygen Saturation (crSO_2_) and Cerebral Fraction of Tissue Oxygen Extraction (FTOE) Values

**Figure 3.**
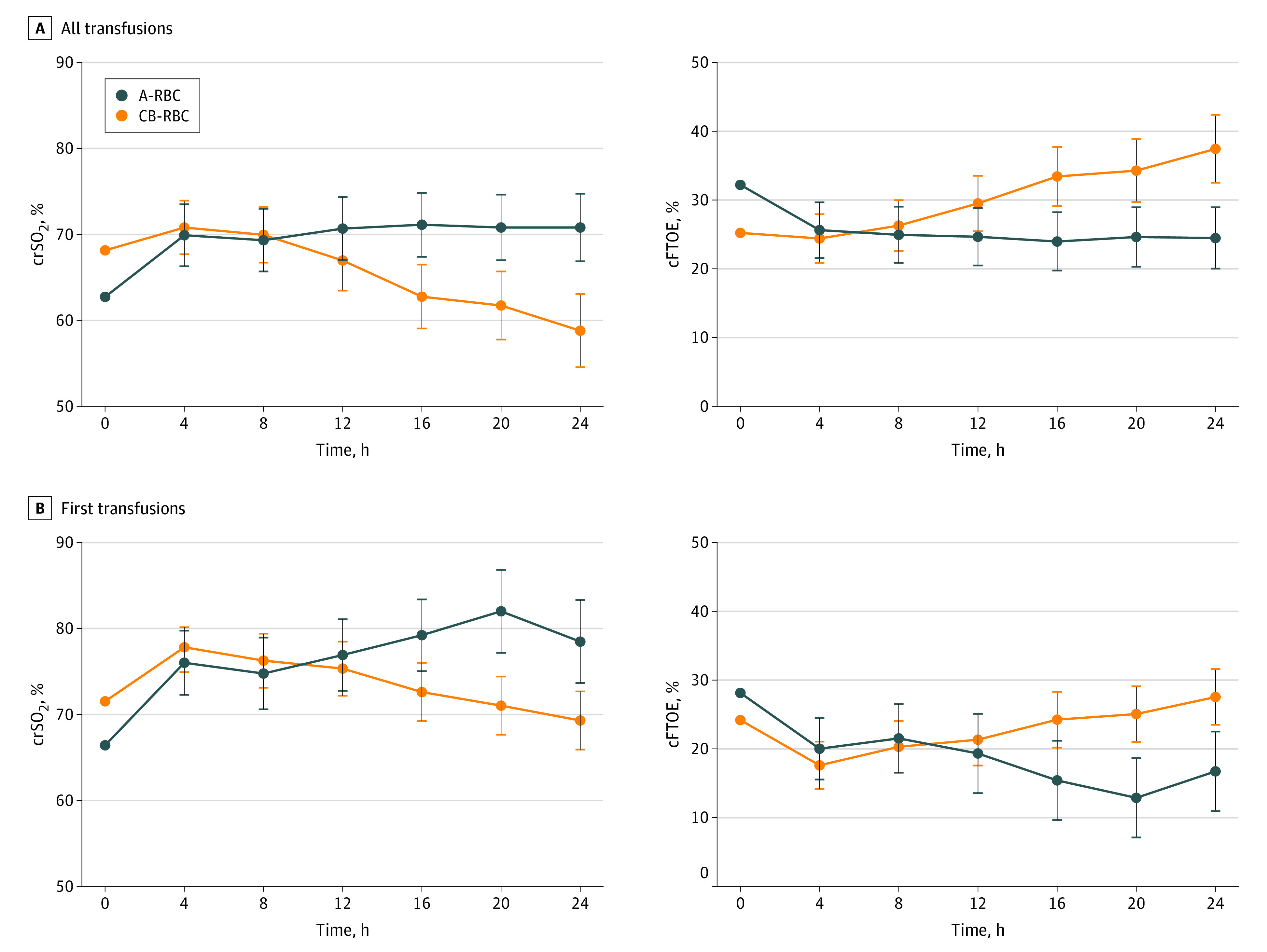
Association of Adult Red Blood Cell (A-RBC) and Cord Blood RBC (CB-RBC) Transfusions With Cerebral Oxygenation Parameters in a Mixed Model for Repeated Measures Analysis cFTOE indicates cerebral fraction of tissue oxygen extraction and crSO_2_, cerebral regional oxygen saturation.

Pretransfusion crSO_2_ and cFTOE values are shown in Table 2. Pretransfusion crSO_2_ before CB-RBCs was slightly higher than it was before A-RBCs (68.2% [IQR, 62.6%-76.2%] vs 62.8% [IQR, 50.5%-67.8%]; *P* = .05). These findings were paralleled by lower but not statistically significant pretransfusion cFTOE (CB-RBC transfusions: 25.2% [IQR, 18.2%-34.8%] vs A-RBC transfusions: 32.2% [IQR, 26.2%-44.0%]; *P* = .07). To explain these differences, we correlated basal oxygenation parameters with postmenstrual age; birth weight; and Hb, Hct, and HbF values at the time of transfusion. The only parameter that showed a weak correlation with basal crSO_2_ (*r* = 0.37; *R*^2^ = 0.13; *P* = .02) and cFTOE (*r* = −0.38; *R*^2^ = 0.14; *P* = .01) was pretransfusion HbF (Figure 2), suggesting that the type of blood component previously received could be involved. Accordingly, when we restricted the analysis exclusively to the first transfusion episodes, we found that pretransfusion values of crSO_2_ and cFTOE were comparable in A-RBC and CB-RBC transfusion events. Results of the MMRM analysis of blood component outcomes on regional oxygenation parameters (crSO2and cFTOE) adjusted for the respective baseline values and for postmenstrual age at transfusion are illustrated in Figure 3. In comparison with A-RBCs, CB-RBCs had a lower posttransfusion covariate-adjusted mean of crSO_2_ of 5.27% (95% CI, 1.20%-9.34%) across all follow-up time points (*P* = .01) (Figure 3A). Considering single intervals, no significant differences were detected between blood products in the first 12 hours after transfusion, while lower crSO_2_ was registered from 16 hours onward after CB-RBC transfusions (eTable 2 in Supplement 1). Conversely, CB-RBCs were associated with a higher cFTOE increase of 6.18% (95% CI, 1.66%-10.69%; *P* = .009) than A-RBCs across all posttransfusion time points (Figure 3A). Considering single intervals, significance was reached at 16, 20, and 24 hours from transfusion (eTable 3 in Supplement 1). To avoid the confounding effect of repeated transfusions, we refined the MMRM analysis considering only the first transfusion episodes (Figure 3B). For both outcomes, differences between blood components became apparent after 12 hours from transfusion (eTables 4 and 5 in Supplement 1). In comparison with nonadjusted models (eTables 6 and 7 in Supplement 1), covariates slightly attenuated the treatment effects. The quality of the selected model is shown in eTable 8 in Supplement 1.

## Discussion

The aim of this cohort study was to assess whether transfusion of a blood product containing HbA or HbF to preterm neonates with anemia was associated with a different oxygen delivery. Our observations suggest that the Hb type was associated with cerebral tissue oxygenation. Repeated transfusions in preterm neonates may cause a progressive and untimely displacement of HbF by HbA, and low HbF levels have been found to be associated with poorer outcome.^8,9,19^ It is arduous to understand whether this occurs because patients who are sicker receive more transfusions or, conversely, whether HbF itself exerts a protective effect from diseases that complicate the clinical course of preterm birth. A difference between HbA and HbF is their oxygen affinity. On the other hand, fragility of prematurity is due primarily to the inability to manage oxidative stress.

First, we found that the HbF reservoir was associated with crSO_2_ and cFTOE in a steady condition of anemia. Neonates with low HbF levels exhibited lower crSO_2_ and higher cFTOE than did neonates with preserved HbF. To our knowledge, there is limited evidence regarding the influence of the HbF level on cerebral oxygenation indexes measured by NIRS.^20^ Previous observations have reported that low HbF in preterm infants was associated with poorer indexes of systemic oxygenation, as measured by median levels of SpO_2_ and partial pressure of carbon dioxide.^21^ Moreover, in accordance with our findings, neonates with higher HbF exhibited higher crSO_2_ and lower cFTOE values during the first 15 minutes after birth.^22^ In contrast, in a case-control study by Wardle et al,^23^ no significant correlation was found between cFTOE and HbF in neonates receiving transfusions (median HbF, 62.5%) and control neonates (median HbF, 84.0%). Additionally, Naulaers et al^24^ measured crSO_2_ in 15 preterm neonates during the first 3 days of postnatal life. The crSO_2_ increased during the 3-day period, but it was unaffected in a multivariate model with changes in HbF; HbF levels conversely decreased during the observation period with A-RBC transfusions (69.7% vs 56.9%). Shorter NIRS records, different types of NIRS equipment, and higher median levels of HbF compared with our cohort could account for the different conclusions in these studies.

The second relevant finding of the present study was the possible contribution of HbF in restoring cerebral oxygenation in parallel with the anemia correction. As expected, both A-RBCs and CB-RBCs were associated with an increase of crSO_2_ and a decrease of cFTOE in the 24 hours following transfusion.^25^ However, after the first 12 hours, the treatment effects on crSO_2_ and cFTOE variation diverged in the 2 different blood components, with lower crSO_2_ and higher cFTOE for CB-RBC compared with A-RBC transfusions. Theoretically, PRBC transfusions in preterm neonates with anemia could influence cerebral oxygenation by 2 primary mechanisms: the increase of arterial oxygen content associated with an increase in Hct and the modification of an HbF-to-HbA ratio. In our study, the first outcome of A-RBC and CB-RBC transfusions was comparable: both products had similar volume and Hct, and both were associated with an equivalent Hct increase. Conversely, A-RBC and CB-RBC transfusions had an opposite outcome with the HbF-to-HbA ratio. We could speculate that in the first 12 hours, the increase of total blood volume and arterial oxygen content may have been salient, thus concealing the differences due to the variation of the HbF-to-HbA ratio and oxygen affinity. Conversely, the right shift of the oxygen dissociation curve after adult but not CB PRBC transfusions could have subsequently produced an increased oxygen delivery. Notably, Naulaers et al^26^ found in a porcine model that in the presence of stable tissue oxygen consumption, the increase of oxygen delivery was associated with a decrease of cFTOE. Thus, among patients in our study, the lower cFTOE observed after A-RBC transfusions may have been associated with a greater oxygen load conveyed by these products to cerebral tissue.^27^

In addition to shedding new light on the response of cerebral parenchyma to PRBC transfusions, these findings may have important clinical implications. Oxidative stress is increasingly recognized as a pathogenic factor for morbidity in preterm neonates without adequate antioxidation capacity.^28^ As an example, in a case-control study, Alderliesten et al^29^ reported higher crSO_2_ and lower cFTOE values in neonates who subsequently developed intraventricular hemorrhage, suggesting hyperperfusion-induced high cerebral tissue oxygenation as a potential causal factor. Interestingly, the intimate anatomical and functional connection between cerebral and retinal vascular beds suggests that cerebral NIRS measurements may also reflect retinal oxygenation kinetics. Accordingly, preterm neonates who develop ROP may spend more time than controls in a condition of cerebral hyperoxia, defined as crSO_2_ greater than 80%.^30^ On the other hand, also in healthy adult volunteers, there is a positive correlation between retinal arterial and venous oxygen saturation measured by spectrophotometric retinal oximetry and crSO_2_ measured by NIRS.^31^ Intriguingly, Vesoulis et al^32^ demonstrated through NIRS monitoring of preterm neonates born before 30 weeks of gestation that low cFTOE but not high SpO_2_ in the first 96 hours was a risk factor for severe ROP. Specifically, neonates who developed severe ROP spent 20% more time than those without severe ROP with cFTOE values less than 15% during a mean recording length of 36 hours, corresponding to an additional 40 minutes of hyperoxia exposure.^32^ We could speculate that the lower levels of cFTOE that we registered after A-RBC transfusions may reflect an increased cerebral oxidative challenge, indirectly indicating an overexposure of retinal tissues to the increased oxygen release by HbA. The inappropriate retinal exposure to oxygen has long been recognized as an important reason for the arrest of vascular growth in ROP.^33^ Furthermore, HbF depletion has been associated with the development of ROP, representing 1 of the potential mechanisms underlying the association between ROP and transfusions.^8,9,34^ The findings of the present study could further support the strategy to transfuse preterm neonates with CB-RBCs to mitigate ROP severity, as this strategy has been proven effective in maintaining the physiological level of HbF.^10^

### Limitations

This study has several limitations. First, due to the small sample size of the investigated population, our findings may not be generalizable and need to be confirmed in larger series of patients. Second, we included in our analysis neonates who received transfusions with both A-RBC and CB-RBC units. Moreover, the NIRS sampling frequency was small compared with other studies. We partially mitigated these limitations by adopting long intervals of NIRS monitoring and a robust MMRM analysis. Finally, we cannot exclude the effects of other unmeasured confounders.

## Conclusions

This cohort study of 17 preterm neonates provides evidence that RBC transfusions from adult donors may be associated with more oxygen delivery to cerebral tissues of preterm neonates than transfusions from CB. This finding may help explain the previously observed connection between low HbF and high ROP risk. It also suggests that use of CB to meet the RBC transfusion needs of neonates with a gestational age of less than 28 weeks may protect cerebral tissues from overexposure to oxygen.

## Notes

Supplement 1. **eAppendix.** SPSS Codes for Adjusted Models **eTable 1.** Types and Progressive Number of RBC Transfusions Received by Each Patient **eTable 2.** Mean Treatment Effect of CB-RBC on crSO_2_ **eTable 3.** Mean Treatment Effect of CB-RBC on cFTOE **eTable 4.** Mean Treatment Effect of CB-RBC on crSO_2_ (First Transfusions) **eTable 5.** Mean Treatment Effect of CB-RBC on cFTOE (First Transfusions) **eTable 6.** Outcome crSO_2_ **eTable 7.** Outcome cFTOE **eTable 8.** AIC Values for Various Covariance Structures

Supplement 2.
Data Sharing Statement
