# Supplementary material for: Differences in Cerebral Tissue Oxygenation in Preterm Neonates Receiving Adult or Cord Blood Red Blood Cell Transfusions
Source: JAMA Netw Open. 2023 Nov 7;6(11):e2341643. doi: 10.1001/jamanetworkopen.2023.41643 (PMC10630897; doi:10.1001/jamanetworkopen.2023.41643)
Supplement: Supplement 1. — eAppendix. SPSS Codes for Adjusted Models eTable 1. Types and Progressive Number of RBC Transfusions Received by Each Patient eTable 2. Mean Treatment Effect of CB-RBC on crSO2 eTable 3. Mean Treatment Effect of CB-RBC on cFTOE eTable 4. Mean Treatment Effect of CB-RBC on crSO2 (First Transfusions) eTable 5. Mean Treatment Effect of CB-RBC on cFTOE (First Transfusions) eTable 6. Outcome crSO2 eTable 7. Outcome cFTOE eTable 8. AIC Values for Various Covariance Structures [file jamanetwopen-e2341643-s001.pdf]

Supplemental Online Content

Pellegrino C, Papacci P, Beccia F, et al. Differences in cerebral tissue oxygenation in preterm neonates receiving adult or cord blood red blood cell transfusions. *JAMA Netw Open*. 2023;6(11):e2341643. doi:10.1001/jamanetworkopen.2023.41643

eAppendix. SPSS Codes for Adjusted Models
eTable 1. Types and Progressive Number of RBC Transfusions Received by Each Patient
eTable 2. Mean Treatment Effect of CB-RBC on $crSO_2$
eTable 3. Mean Treatment Effect of CB-RBC on cFTOE
eTable 4. Mean Treatment Effect of CB-RBC on $crSO_2$ (First Transfusions)
eTable 5. Mean Treatment Effect of CB-RBC on cFTOE (First Transfusions)
eTable 6. Outcome $crSO_2$
eTable 7. Outcome cFTOE
eTable 8. AIC Values for Various Covariance Structures

This supplemental material has been provided by the authors to give readers additional information about their work.

© 2023 Pellegrino C et al. *JAMA Network Open*.

eAppendix. SPSS Codes for Adjusted Models

```
MIXED CrSO2 BY replication trt time id WITH CrSO2_baseline PMAattransfusion

  /CRITERIA=DFMETHOD(SATTERTHWAITE) CIN(95) MXITER(100) MXSTEP(10) SCORING(1)
SINGULAR(0.000000000001) HCONVERGE(0, ABSOLUTE) LCONVERGE(0, ABSOLUTE)

  PCONVERGE(0.000001, ABSOLUTE)

  /FIXED=replication trt time trt*time CrSO2_baseline PMAattransfusion | SSTYPE(3)

  /METHOD=ML

  /RANDOM=INTERCEPT | SUBJECT(id) COVTYPE(VC)

  /REPEATED=replication*time | SUBJECT(id) COVTYPE(AR1)

  /EMMEANS=TABLES(replication) COMPARE ADJ(LSD)

  /EMMEANS=TABLES(trt) COMPARE ADJ(LSD)

  /EMMEANS=TABLES(time) COMPARE ADJ(LSD)

  /EMMEANS=TABLES(trt*time) COMPARE (trt) ADJ(LSD)


MIXED FTOE BY replication trt time id WITH FTOE_baseline PMAattransfusion

  /CRITERIA=DFMETHOD(SATTERTHWAITE) CIN(95) MXITER(100) MXSTEP(10) SCORING(1)
SINGULAR(0.000000000001) HCONVERGE(0, ABSOLUTE) LCONVERGE(0, ABSOLUTE)

  PCONVERGE(0.000001, ABSOLUTE)

  /FIXED=replication trt time trt*time FTOE_baseline PMAattransfusion | SSTYPE(3)

  /METHOD=ML

  /RANDOM=INTERCEPT | SUBJECT(id) COVTYPE(VC)

  /REPEATED=replication*time | SUBJECT(id) COVTYPE(AR1)

  /EMMEANS=TABLES(replication) COMPARE ADJ(LSD)

  /EMMEANS=TABLES(trt) COMPARE ADJ(LSD)

  /EMMEANS=TABLES(time) COMPARE ADJ(LSD)

  /EMMEANS=TABLES(trt*time) COMPARE (trt) ADJ(LSD)
```

© 2023 Pellegrino C et al. *JAMA Network Open*.

eTable 1. Types and Progressive Number of RBC Transfusions Received by Each Patient

Patient ID	Transfusion ID	Progressive number	PRBC type
A	A1	1	CB-RBC
B	B1	1	A-RBC
	B2	2	A-RBC
	B3	3	A-RBC
	B4	4	A-RBC
C	C1	1	CB-RBC
	C2	2	A-RBC
	C3	3	CB-RBC
	C4	4	CB-RBC
D	D1	1	CB-RBC
	D2	2	CB-RBC
E	E1	1	A-RBC
F	F1	1	A-RBC
	F2	3	A-RBC
	F3	4	A-RBC
G	G1	2	A-RBC
	G2	3	A-RBC
	G3	4	A-RBC
	G4	5	A-RBC
H	H1	6	CB-RBC
	H2	7	CB-RBC
I	I1	1	A-RBC
	I2	4	A-RBC
J	J1	1	A-RBC
	J2	3	A-RBC
K	K1	1	CB-RBC
	K2	2	CB-RBC
	K3	3	A-RBC
	K4	4	A-RBC
	K5	5	A-RBC

© 2023 Pellegrino C et al. *JAMA Network Open*.

	K6	6	CB-RBC
	K7	7	CB-RBC
L	L1	1	A-RBC
M	M1	1	CB-RBC
N	N1	1	CB-RBC
O	O1	1	A-RBC
P	P1	1	CB-RBC
Q	Q1	1	CB-RBC
	Q2	2	CB-RBC
	Q3	3	CB-RBC
	Q4	5	CB-RBC
	Q5	6	CB-RBC

© 2023 Pellegrino C et al. *JAMA Network Open*.

eTable 2. Mean Treatment Effect of CB-RBC on $CrSO_2$

Decrease of 5.269 (95% CI: 1,200-9,339)

**Treatment**	0.013
**Time**	0,008
**Treatment* Time**	<0,001
**$CrS0_2$ Baseline**	<0,001
**PMA at transfusion**	0,001
**Intercept**	0,076
**Replication**	0,023

RBC unit type * time: Mean treatment effects at each follow-up timepoint

Estimates

**Time Interval**	Mean difference **A-RBC – C-RBC**	**Standard error**	**df**	**Significance**	**95% C.I. Lower Bound**	**95% C.I. Upper bound**
**0- 4 h**	- 0,902	2,224	55,678	0,687	-5,357	3,554
**4-8 h**	- 0,614	2,309	61.836	0,791	-5,229	4,001
**8-12 h**	3,714	2,406	66.468	0,127	-1,089	8,516
**12-16 h**	8,348	2,507	69,625	0.001	3,348	13,349
**16-20 h**	9,077	2,647	74,491	< 0.001	3,803	14,350
**20-24 h**	11.922	2,769	75,808	< 0.001	6,477	17,508

Pairwise comparison

**RBC unit type**	**Time**	**Mean**	**Standard error**	**df**	**95% Confidence Interval**	
					**Lower Bound**	**Upper Bound**
A-RBC	**0- 4 h**	69,918	1,799	52,500	66,309	73,527
	**4-8 h**	69,342	1,818	54,181	65,697	72,988
	**8-12 h**	70,685	1,829	54,404	67,019	74,352
	**12-16 h**	71,129	1,857	56,192	67,409	74,849
	**16-20 h**	70,822	1,914	60,224	66,994	74,650
	**20-24 h**	70,813	1,970	62,327	66,876	74,750
CB-RBC	**0- 4 h**	70,820	1,556	56,560	67,704	73,935
	**4-8 h**	69,956	1,618	63,582	66,724	73,189
	**8-12 h**	66,972	1,754	75,382	63,478	70,465
	**12-16 h**	62,780	1,868	79,843	59,064	66,497
	**16-20 h**	61,745	1,990	83,052	57,788	65,703
	**20-24 h**	58,821	2,141	85,599	54,565	63,076

© 2023 Pellegrino C et al. *JAMA Network Open*.

eTable 3. Mean Treatment Effect of CB-RBC on cFTOE

Increase of 6.175 (95% CI: 1,662-10,687)

**Treatment**	0.009
**Time**	0,026
**Treatment* Time**	0.002
**FTOE Baseline**	<0,001
**PMA at transfusion**	0,007
**Intercept**	0,005
**Replication**	0,077

RBC unit type * time: Mean treatment effects at each follow-up timepoint

Estimates

RBC unit type	Time	Mean	Standard error	df	95% Confidence Interval	
					Lower Bound	Upper Bound
A-RBC	**0- 4 h**	25,640	2,024	56,766	21,586	29,693
	**4-8 h**	24,965	2,049	58,723	20,865	29,064
	**8-12 h**	24,672	2,087	61,687	20,499	28,845
	**12-16 h**	23,992	2,125	64,040	19,747	28,237
	**16-20 h**	24,647	2,168	65,320	20,317	28,977
	**20-24 h**	24,492	2,235	67,159	20,031	28,954
CB-RBC	**0- 4 h**	24,416	1,774	60,955	20,869	27,963
	**4-8 h**	26,305	1,855	69,101	22,604	30,006
	**8-12 h**	29,516	2,025	81,312	25,487	33,546
	**12-16 h**	33,451	2,161	84,431	29,154	37,747
	**16-20 h**	34,303	2,305	86,560	29,720	38,886
	**20-24 h**	37,465	2,483	88,144	32,531	42,399

Pairwise comparison

Time Interval	MEAN DIFFERENCE A-RBC – C-RBC	Standard error	df	Significance	95% C.I. Lower Bound	95% C.I. Upper bound
**0- 4 h**	1,224	2,521	60,449	0,629	-3,817	6,265
**4-8 h**	-1,341	2,630	67,529	0,612	-6,588	3,907
**8-12 h**	-4,844	2,772	74,363	0,085	-10,367	,680
**12-16 h**	-9,459	2,893	77,014	0,002	-15,220	-3,697
**16-20 h**	-9,656	3,038	79,088	0,002	-15,703	-3,609
**20-24 h**	-12,973	3,181	79,685	<0,001	-19,304	-6,642

© 2023 Pellegrino C et al. *JAMA Network Open*.

eTable 4. Mean Treatment Effect of CB-RBC on $CrSO_2$ (First Transfusions)

Decrease of 4.174 (95% CI: 1,531-6,817)

**Treatment**	0.003
**Time**	0,693
**Treatment* Time**	0.002
**$CrS0_2$ Baseline**	<0,001
**PMA at transfusion**	0,002
**Intercept**	0,423

RBC unit type * time: Mean treatment effects at each follow-up timepoint

Estimates

RBC unit type	Time	Mean	Standard error	df	95% Confidence Interval	
					Lower Bound	Upper Bound
A-RBC	**0- 4 h**	76,025	1,863	49	72,281	79,768
	**4-8 h**	74,784	2,074	49	70,616	78,952
	**8-12 h**	76,931	2,074	49	72,763	81,100
	**12-16 h**	79,227	2,074	49	75,059	83,395
	**16-20 h**	82,009	2,404	49	77,178	86,840
	**20-24 h**	78,492	2,404	49	73,661	83,323
CB-RBC	**0- 4 h**	77,825	1,436	49	74,940	80,710
	**4-8 h**	76,270	1,561	49	73,132	79,408
	**8-12 h**	75,342	1,561	49	72,204	78,480
	**12-16 h**	72,631	1,685	49	69,244	76,018
	**16-20 h**	71,046	1,685	49	67,659	74,433
	**20-24 h**	69,311	1,685	49	65,924	72,698

Pairwise comparison

Time Interval	MEAN DIFFERENCE A-RBC – C-RBC	Standard error	df	Significance	95% C.I. Lower Bound	95% C.I. Upper bound
**0- 4 h**	-1,801	2,369	49	0,451	-6,562	2,961
**4-8 h**	-1,486	2,648	49	0,577	-6,808	3,835
**8-12 h**	1,590	2,648	49	0,551	-3,732	6,911
**12-16 h**	6,596	2,726	49	0,019	1,117	12,075
**16-20 h**	10,963	2,996	49	<,001	4,942	16,984
**20-24 h**	9,181	2,996	49	0,004	3,159	15,202

© 2023 Pellegrino C et al. *JAMA Network Open*.

eTable 5. Mean Treatment Effect of CB-RBC on cFTOE (First Transfusions)

Increase of 5.031 (95% CI:1,779-8,284)

**Treatment**	0.003
**Time**	0,692
**Treatment* Time**	0.004
**FTOE Baseline**	<0,001
**PMA at transfusion**	0,016
**Intercept**	0,006

RBC unit type * time: Mean treatment effects at each follow-up timepoint

Estimates

RBC unit type	Time	Mean	Standard error	df	95% Confidence Interval	
					Lower Bound	Upper Bound
A-RBC	**0- 4 h**	20,026	2,228	47	15,544	24,508
	**4-8 h**	21,531	2,479	47	16,543	26,519
	**8-12 h**	19,344	2,871	47	13,568	25,121
	**12-16 h**	15,428	2,871	47	9,651	21,204
	**16-20 h**	12,895	2,873	47	7,115	18,676
	**20-24 h**	16,749	2,873	47	10,969	22,530
CB-RBC	**0- 4 h**	17,615	1,716	47	14,163	21,067
	**4-8 h**	20,310	1,865	47	16,557	24,063
	**8-12 h**	21,350	1,865	47	17,597	25,103
	**12-16 h**	24,252	2,013	47	20,203	28,301
	**16-20 h**	25,071	2,013	47	21,022	29,120
	**20-24 h**	27,562	2,013	47	23,513	31,611

Pairwise comparison

Time Interval	MEAN DIFFERENCE A-RBC – C-RBC	Standard error	df	Significance	95% C.I. Lower Bound	95% C.I. Upper bound
**0- 4 h**	2,410	2,829	47	0,399	-3,281	8,102
**4-8 h**	1,221	3,161	47	0,701	-5,139	7,580
**8-12 h**	-2,006	3,491	47	0,568	-9,029	5,017
**12-16 h**	-8,825	3,572	47	0,017	-16,012	-1,638
**16-20 h**	-12,176	3,577	47	0,001	-19,373	-4,979
**20-24 h**	-10,813	3,577	47	0,004	-18,010	-3,616

© 2023 Pellegrino C et al. *JAMA Network Open*.

Sensitivity analysis. Mean treatment effect estimates after removing first transfusions from the dataset – multiple transfusions analysis.

eTable 6. Outcome $crSO_2$

Effect of treatment: Decrease of 10.363 (95%CI: 5.198 – 15.528), P<0.001
Treatment x time interaction: P<0.001

Pairwise Comparisons[a]

time	(I) RBC unit type	(J) RBC unit type	Mean Difference (I-J)	Std. Error	df	Sig.[e]	95% Confidence Interval for Difference[e]	
							Lower Bound	Upper Bound
4	adult	cordonal	.653	2.620	30.264	.805	-4.696	6.001
	cordonal	adult	-.653	2.620	30.264	.805	-6.001	4.696
8	adult	cordonal	.306	2.763	35.504	.912	-5.300	5.912
	cordonal	adult	-.306	2.763	35.504	.912	-5.912	5.300
12	adult	cordonal	8.391*	3.053	43.382	.009	2.236	14.546
	cordonal	adult	-8.391*	3.053	43.382	.009	-14.546	-2.236
16	adult	cordonal	13.965*	3.212	42.635	<.001	7.485	20.444
	cordonal	adult	-13.965*	3.212	42.635	<.001	-20.444	-7.485
20	adult	cordonal	13.610*	3.643	51.516	<.001	6.299	20.921
	cordonal	adult	-13.610*	3.643	51.516	<.001	-20.921	-6.299
24	adult	cordonal	26.424*	4.397	62.230	<.001	17.635	35.213
	cordonal	adult	-26.424*	4.397	62.230	<.001	-35.213	-17.635
28	adult	cordonal	.[c]	.	.	.	.	.
	cordonal	adult	.[d]	.	.	.	.	.

Based on estimated marginal means
*. The mean difference is significant at the .05 level.

a. Dependent Variable: sO2.

c. The level combination of factors in (J) is not observed.

d. The level combination of factors in (I) is not observed.

e. Adjustment for multiple comparisons: Least Significant Difference (equivalent to no adjustments).

© 2023 Pellegrino C et al. *JAMA Network Open*.

eTable7. Outcome cFTOE

Effect of treatment: Increase of 11.635 (95%CI: 5.5503 – 17.767), P<0.001

Treatment x time interaction: P<0.001

Pairwise Comparisons[a]

time	(I) RBC unit type	(J) RBC unit type	Mean Difference (I-J)	Std. Error	df	Sig.[c]	95% Confidence Interval for Difference[c] Lower Bound	Upper Bound
4	adult	cordonal	-1,093	3,115	30,963	,728	-7,448	5,261
	cordonal	adult	1,093	3,115	30,963	,728	-5,261	7,448
8	adult	cordonal	-3,495	3,288	36,087	,295	-10,162	3,172
	cordonal	adult	3,495	3,288	36,087	,295	-3,172	10,162
12	adult	cordonal	-9,939*	3,628	43,351	,009	-17,253	-2,624
	cordonal	adult	9,939*	3,628	43,351	,009	2,624	17,253
16	adult	cordonal	-14,328*	3,806	42,300	<,001	-22,008	-6,648
	cordonal	adult	14,328*	3,806	42,300	<,001	6,648	22,008
20	adult	cordonal	-15,072*	4,339	50,712	,001	-23,784	-6,359
	cordonal	adult	15,072*	4,339	50,712	,001	6,359	23,784
24	adult	cordonal	-25,885*	5,263	60,063	<,001	-36,412	-15,358
	cordonal	adult	25,885*	5,263	60,063	<,001	15,358	36,412

Based on estimated marginal means

*. The mean difference is significant at the ,05 level.

a. Dependent Variable: FTOE.

c. Adjustment for multiple comparisons: Least Significant Difference (equivalent to no adjustments).

© 2023 Pellegrino C et al. *JAMA Network Open*.

eTable 8. AIC Values for Various Covariance Structures

The AR1 covariance structure was selected for models on crSO2 and FTOE outcomes on the basis of the smaller is better AIC approach (Ferron et al., 2002)

Ferron J, Dailey R, Yi Q. Effects of Misspecifying the First-Level Error Structure in Two-Level Models of Change. Multivariate Behav Res. 2002 Jul 1;37(3):379-403

**Covariance structure**	**Akaike's Information Criteria Statistic**	
	**cFTOE**	**$CrS0_2$**
Compound Symmetry	1023.107	954.411
1st Order autoregressive	**962.847**	**887.690**
Diagonal	989.921	945.106
Scaled Identity	1021.107	952.411
Unstructured	Not fitted	Not fitted
Correlated Compound Symmetry	1023.107	966.472
Compound Symmetry Heterogenous	989.624	1034.797

© 2023 Pellegrino C et al. *JAMA Network Open*.
