# Supplementary material for: Differences in Cerebral Tissue Oxygenation in Preterm Neonates Receiving Adult or Cord Blood Red Blood Cell Transfusions
Source: JAMA Netw Open. 2023 Nov 7;6(11):e2341643. doi: 10.1001/jamanetworkopen.2023.41643 (PMC10630897; doi:10.1001/jamanetworkopen.2023.41643)
Supplement: Supplement 2. — Data Sharing Statement [file jamanetwopen-e2341643-s002.pdf]

Data Sharing Statement

Data
Data available: Yes
Data types: Deidentified participant data
How to access data: Data will be available upon request to the corresponding author.
When available: With publication

Supporting Documents
Document types: Statistical/analytic code
How to access documents: Statistical and analytic codes are provided as supplementary material.
When available: With publication

Additional Information
Who can access the data: data will be made available to researchers whose proposed use of the data has been approved
Types of analyses: the data will be made available only for research purposes
Mechanisms of data availability: the data will be made available with investigator support after approval of a proposal and with a signed data access agreement
